# Listen, act and support: An investigation into individual and organisational incivility management in veterinary practice

**DOI:** 10.1002/vetr.4840

**Published:** 2025-01-07

**Authors:** Amy Irwin, Luiz Santos, Helen Silver‐MacMahon, Liz Mossop

**Affiliations:** ^1^ Applied Psychology & Human Factors Research Group School of Psychology University of Aberdeen Aberdeen UK; ^2^ School of Biodiversity, One Health & Veterinary Medicine University of Glasgow Glasgow UK; ^3^ Being Human Consulting London UK; ^4^ Sheffield Hallam University Sheffield UK

## Abstract

**Background:**

This study investigates the impact of workplace and client incivility on veterinary staff wellbeing and job satisfaction, examining both individual responses and organisational support mechanisms to identify best practices for managing incivility.

**Method:**

A mixed‐methods approach was employed, involving a survey of 192 veterinary professionals from various roles and practice types. The survey measured experiences of incivility, individual factors (anxiety, stress, burnout, job satisfaction and turnover intention) and organisational factors (perceived organisational support, social support and civility climate).

**Results:**

Client incivility was a significant predictor of increased anxiety, burnout and stress, while co‐worker incivility was a significant predictor of increased anxiety. Organisational support and team civility were found to be significant predictors of job satisfaction and turnover intention, with organisational support mediating the impact of co‐worker incivility on anxiety. Qualitative analysis highlighted the importance of listening to staff concerns and taking proactive measures to address incivility.

**Limitations:**

The data are cross‐sectional and subjective, and the sample is predominantly female.

**Conclusion:**

Effective management of incivility in veterinary practices requires robust organisational support and clear policies. Practices should prioritise listening to staff, fostering a supportive environment and implementing training programs to mitigate the adverse effects of incivility on staff wellbeing and job satisfaction.

## INTRODUCTION

Workplace incivility is generally defined as insensitive or disrespectful behaviour that can be ambiguous in intent and violates workplace expectations of civility.^1^ Incivility has consistently been found to adversely affect the cognitive, emotional and behavioural aspects of those who experience or witness this form of behaviour.^1^, ^2^ Research also indicates a potential negative impact on veterinary staff, with reports of reduced self‐confidence, negative mood and increased anxiety.^3^, ^4^, ^5^ More specifically, incivility from veterinary clients has been linked to increased depression and burnout, while co‐worker incivility has been associated with decreased job satisfaction and increased quitting intentions.^6^

### Managing incivility

Although extensive research has been conducted on workplace incivility,^7^ there is a gap in understanding individual responses and the role of organisational factors. Studies suggest that targets often ignore incivility due to its ambiguity.^8^ Recent research on coping strategies revealed that neither passive avoidance nor active confrontation effectively reduced future incivility. Avoidance was linked to higher emotional exhaustion and an increased likelihood of the target becoming uncivil.^9^ In contrast, confrontation was linked to psychological forgiveness of the perpetrator, suggesting that this response may lead to some form of closure.^9^ A further study explored problem‐focused (active and planning) and emotion‐focused (avoidance, religious and support seeking) coping mechanisms in response to incivility,^10^ with the latter weakening the relationship between job satisfaction and incivility (meaning incivility was less likely to adversely impact satisfaction), making it more effective as a response.

Cortina et al.^11^ developed a biobehavioural model of responses to incivility, split into four quadrants: reciprocation, retreat, relationship repair and recruitment of support. The model was used as the basis for a study examining veterinary staff responses to incivility vignettes.^12^ The results indicated that veterinarians and veterinary nurses alter their response to incivility depending on their appraisal of the behaviour and the form of incivility depicted. In other words, responses to incivility are tailored to the situation and can vary across different forms of incivility. However, although this research is indicative of individual responses to incivility, the findings do not consider organisational‐level factors or support.

Research examining the relationship between organisational factors and workplace incivility is relatively sparse, but what does exist indicates that multiple aspects of an organisation could influence the frequency and impact of incivility. Within education, research suggests that an individual is more likely to be uncivil during periods of organisational change, where their job is insecure and they lack support from their colleagues, and in response to incivility from co‐workers.^13^ Research investigating potential links between team climate, norms and incivility impacts reported that an uncivil team climate was associated with reduced employee wellbeing and that competitive team norms moderated the impact of experienced incivility by reducing the negative consequences for individual wellbeing.^14^ (Competitive team norms relate to the extent to which workers feel their success is compared to others – in a competitive environment they will feel that to do well they will need to do better than their peers) Finally, qualitative findings across incivility research within the veterinary context^3^, ^12^ have consistently highlighted the perceived importance of practice culture and support mechanisms for the ability of individuals to both manage incivility and mitigate any adverse consequences.

As this body of research continues to grow, it seems apparent that incivility is an important workplace stressor within the veterinary context. We suggest that the next step in this research is to examine individual strategies and organisational support mechanisms to identify best practice for managing incivility.

### Study aims

This study aimed to replicate and expand on previous research examining incivility within the veterinary context, with a specific focus on individual responses and organisational support mechanisms. The research questions guiding this study were as follows:

- R1: Are individual factors (anxiety, stress, burnout, job satisfaction and turnover intention) associated with incivility (client or co‐worker) or organisational factors (organisational support, social support and civility climate)?
- R2: Is the relationship between incivility (client or co‐worker) and individual factors mediated by organisational factors?
- R3: Is there an association between incivility response type and individual or organisational factors?
- R4: What are the current practice‐based approaches to managing incivility, and how could these approaches be enhanced?

## METHOD

### Participants

A total of 192 participants (169 female [88%], 15 male [8%], two non‐binary [1%], six prefer not to say [4%]; mean age 41 years) were gathered from around the world (UK [*n* = 150; 78%], Europe [*n* = 12; 6%], Australia and New Zealand [*n* = 12; 6%], USA and Canada [*n* = 6; 4%] and not stated [*n* = 12; 6%]) via convenience sampling. The participants needed to be aged 18 years or over and work full‐time in a veterinary practice. Participants were recruited via social media (including veterinary Facebook groups, X and LinkedIn), direct email invitation (via veterinary contacts known to the authors and using the listed email contacts of veterinary practices on the Royal College of Veterinary Surgeons website) and via forwarded messages through large organisations (e.g., the Veterinary Management Group). The sample comprised veterinarians (*n* = 86; 45%), veterinary nurses (*n* = 38; 20%), practice managers (*n* = 16; 8%), animal care assistants (*n* = 6; 3%), receptionists (*n* = 36; 19%) and not stated (*n* = 10; 5%). The participants predominantly worked in small animal practices (*n* = 150; 78%) with the remainder working in mixed (*n* = 19; 10%), large animal (*n* = 4; 2%), equine (*n* = 13; 7%), exotic (*n* = 1; 0.5%) and other (*n* = 5; 5.5%).

### Questionnaire

The online questionnaire consisted of four main sections and was created using SNAP survey software (www.snapsurveys.com).

Section 1: The first section comprised questions relevant to demographic information; encompassing job role, gender, age, country of residence and practice focus.

Section 2: This section was split into two sub‐sections:

Sub‐section A comprised the short workplace incivility scale (WIS),^15^ encompassing four statements, for example, ‘Paid little attention to your statements or showed little interest in your opinions’, where participants indicated the frequency with which they had experienced the stated behaviour (from 1—never, to 5—many times). The initial statement for the scale was edited to focus on client incivility: ‘During the past 6 months were you ever in a situation in which a CLIENT within your practice’. This was followed by 12 items, each reflective of a specific form of response (from 1—never, to 5—very often). Eight of these items have been used previously in veterinary research^12^ and represent the response options of ignoring the incivility, confronting the behaviour, talking to a friend or colleague about it, reporting the behaviour to a senior colleague, trying to avoid the person following the interaction, making a friendly overture, reciprocation and exit. These were combined with an additional four items that were designed to reflect responses to incivility reported across two veterinary incivility studies^6^, ^12^: involve another colleague, remain calm, focus on the task at hand and consider the underlying reasons for behaviour. Each item depicts a specific response, so these items do not attempt to measure an underlying construct.

Sub‐section B comprised the same scales and items, but here the initial statement of the WIS scale^15^ was altered to assess the level of, and responses towards, co‐worker incivility.

Section 3: The aim of this section was to gather information on participant anxiety, stress, burnout, job satisfaction and turnover intention. The participants were asked to indicate their current level of anxiety using the Generalised Anxiety Disorder‐7 item scale (GAD‐7) anxiety index.^16^ The index asks participants the extent to which they have felt bothered with different aspects of anxiety, for example, ‘Feeling nervous, anxious or on edge’ (from 0—not at all, to 3—every day). The participants were then asked to report their current level of job satisfaction using the single item, ‘Taking everything into consideration, how do you feel about your job as a whole? (from 1—extremely dissatisfied, to 7—extremely satisfied).^17^ Next, the questionnaire included a three‐item measure of turnover intention (e.g., ‘I have been actively looking for other jobs’, from 1—strongly disagree, to 5—strongly agree).^18^ The participants were then asked to complete a one‐item measure of stress: ‘In the past 6 months, how would you rate the amount of stress in your life (at work and at home)’ (1—no stress, to 6—extreme stress).^19^ Finally, the participants were asked to complete a single‐item assessment of burnout level; ‘I feel burned out from my work’ (1—never, to 7—daily).^20^

Section 4: The final section comprised three measures relevant to support in the workplace. First, the participants completed the eight‐item version of the survey of perceived organisational support (SPOS), which is designed to assess the extent to which workers perceive their workplace to care about their wellbeing and provide support (e.g., ‘my veterinary practice really cares about my wellbeing', from 1—strongly agree, to 7—strongly disagree).^21^ This was followed by the four‐item team civility scale (e.g., ‘my team treats one another with respect’, from 1—strongly agree, to 5—strongly disagree).^22^ Finally, the participants completed the brief perceived social support scale (PSSS), which provides an indication of support and positive interactions perceived within the workplace (e.g., ‘I experience a lot of understanding and security from colleagues at work’, from 1—not true at all, to 5—very true).^23^ The scales were followed by two open‐ended questions: ‘What does your veterinary practice do currently to manage incivility and/or support staff experiencing incivility?’ and ‘What do you consider to be the best way in which veterinary practices can support their staff in managing incivility?’.

### Data analysis

#### Quantitative

Research questions 1 and 3 were investigated using Pearson bivariate correlation followed by multiple regression analysis. Research question 2 was addressed using mediation analysis.

#### Qualitative

Research question 4 was addressed using conventional content analysis,^24^ with coding developed based on the semantic content provided in response to two open‐ended questions. Coding was completed by the first author via three phases. Phase one involved reading the questionnaire responses several times to enhance familiarity with the content. In phase two, each response was then coded to reflect the semantic content of the response, with a focus on the nature of the described practice‐based incivility management strategy. Finally, codes were organised into categories based on shared meaning, with each category named to reflect a form of strategy.

## RESULTS

### Descriptive overview

The scale scores represent the summed score generated by combining items. The participants were free to skip any items within the questionnaire that they did not wish to answer; as such, some of the participants had missing data points for some of the scales. Where this occurred, the summed score was removed from the analysis, which led to the loss of 25 scale scores. Table 1 presents the mean scores, with standard deviations, for all the key scales and variables. Prior to conducting the statistical analyses, the key scale‐based variables (anxiety, turnover intention, workplace incivility, perceived organisational support, team civility climate and perceived social support) were evaluated for scale reliability with an alpha requirement of 0.7 or above for inclusion in further analysis (Table 2). In addition, skewness and kurtosis values were checked with the supposition that values that fall within ±2 are considered within the acceptable range for analysis. As a result, the response items ‘reciprocate’ and ‘task focus’ were removed from further analysis.

**TABLE 1:** Mean scores and standard deviations for individual and organisational variables.

Variable	Score range	Mean (SD)	Skewness	Kurtosis	Scale reliability (Cronbach's alpha)
Job satisfaction	1‒7	4.72 (1.74)	‒0.677	‒0.749	
Burnout	1‒7	4.48 (1.30)	‒0.199	0.166	
Stress	2‒6	4.44 (1.02)	‒0.305	‒0.213	
Turnover intention	3‒15	7.44 (3.79)	0.448	‒0.946	0.76
GAD‐7 anxiety scale	7‒28	16.26 (5.67)	0.377	‒0.591	0.92
Perceived organisational support scale	8‒56	36.08 (11.76)	‒0.318	‒0.690	0.90
Team civility climate scale	4‒20	13.96 (3.64)	‒0.445	‒0.281	0.85
Perceived social support scale	9‒30	22.15 (4.75)	‒0.260	‒0.392	0.86

**TABLE 2:** Mean scores and standard deviations for workplace incivility and response variables.

		Client	Client skewness	Client kurtosis	Co‐worker	Co‐worker skewness	Co‐worker kurtosis
Incivility level (workplace incivility scale)	4‒20	10.19 (3.51)	0.538	0.288	10.74 (4.24)	0.276	‒0.642
Friendly overture	1‒5	3.60 (1.05)	‒0.486	‒0.031	3.21 (1.09)	‒0.172	‒0.442
Ignore	1‒5	3.64 (1.01)	‒0.513	‒0.040	3.64 (1.02)	‒0.489	‒0.161
Confront the behaviour	1‒5	2.34 (1.03)	0.657	0.098	2.59 (1.07)	0.293	‒0.451
Reciprocate	1‒4	1.35 (0.63)	2.004	4.300	1.78 (0.92)	0.950	0.164
Leave the situation	1‒5	2.38 (0.97)	0.437	‒0.023	3.30 (0.96)	‒0.255	0.156
Avoid that person	1‒5	2.93 (1.17)	0.057	‒0.648	3.13 (1.25)	‒0.198	‒0.916
Talk to friend/colleague	1‒5	4.03 (0.91)	‒0.740	0.397	3.63 (1.14)	‒0.579	‒0.343
Involve a colleague	1‒5	2.67 (1.19)	0.228	‒0.794	2.24 (1.13)	0.610	‒0.438
Report/discuss with senior staff	1‒5	3.21 (1.21)	‒0.154	‒0.844	2.81 (1.25)	0.148	‒0.996
Emotion management	1‒5	4.05 (0.89)	‒1.076	1.641	3.72 (1.01)	‒0.673	0.388
Focus on task	1‒5	4.29 (0.72)	‒1.130	2.369	4.02 (0.87)	‒1.029	1.660
Consider reasons for behaviour	1‒5	3.76 (1.03)	‒0.474	‒0.396	3.53 (1.08)	‒0.336	‒0.345

### R1: Links between incivility, individual factors and organisational factors

The next step in the analysis was to determine whether there were any associations between incivility (client and co‐worker), individual factors (stress, anxiety, burnout, job satisfaction and turnover intention) and organisational factors (perceived organisational support, perceived social support and team civility climate). To assess this, a Pearson bivariate correlation matrix was produced (Table 3). The results indicate that client incivility was positively correlated with burnout, stress and anxiety. Co‐worker incivility was negatively correlated with job satisfaction, organisational support, team civility and social support, and positively correlated with burnout, stress, turnover and anxiety. Organisational support, team civility and social support were all positively correlated with job satisfaction, and negatively correlated with burnout, stress, turnover and anxiety.

**TABLE 3:** Bivariate correlations between incivility, measures of impact (job satisfaction, stress, anxiety and turnover) and measures of support (organisational support, social support and team incivility)

Variable	1.	2.	3.	4.	5.	6.	7.	8.	9.
1. Client incivility	^—^								
2. Co‐worker incivility	**0.182** ^*^	^—^							
3. Job satisfaction	‒0.052	**‒0.234** ^**^	^—^						
4. Burnout	**0.288** ^**^	**0.340** ^**^	**‒0.596** ^**^	^—^					
5. Stress	**0.189** ^*^	**0.331** ^**^	**‒0.451** ^**^	**0.690** ^**^	^—^				
6. Turnover	0.026	**0.379** ^**^	**‒0.700** ^**^	**0.568** ^**^	**0.420** ^**^	^—^			
7. Anxiety	**0.225** ^**^	**0.416** ^**^	**‒0.447** ^**^	**0.585** ^**^	**0.710** ^**^	**0.413** ^**^	^—^		
8. Organisational support	‒0.052	**‒0.458** ^**^	**0.589** ^**^	**‒0.471** ^**^	**‒0.363** ^**^	**‒0.620** ^**^	**‒0.434** ^**^	^—^	
9. Team civility	**‒0.052**	**‒0.540** ^**^	**0.486** ^**^	**‒0.393** ^**^	**‒0.290** ^**^	**‒0.555** ^**^	**‒0.337** ^**^	**0.650** ^**^	^—^
10. Social support	**‒0.014**	**‒0.313** ^**^	**0.372** ^**^	**‒0.309** ^**^	**‒0.277** ^**^	**‒0.459** ^**^	**‒0.347** ^**^	**0.545** ^**^	**0.544** ^**^

*Note*: Bold text signifies significant results, with asterisks indicating the degree of significance.

^*^ *p* < 0.05.

^**^ *p* < 0.005.

Next, a series of five multiple regression analyses, with all potential predictors entered simultaneously in each analysis, were conducted to determine if incivility (client and co‐worker) and organisational factors (perceived organisational support, team civility and social support) functioned as predictors of individual factors (burnout, stress, anxiety, job satisfaction and turnover intention). The results (Table 4) indicated several significant regression equations, which will be described in turn below.

**TABLE 4:** Multiple linear regression analysis to determine the extent to which incivility and organisational factors function as predictors of employee wellbeing and satisfaction

Variable	*B*	SE	β	*T*	*p*‐value
Dependent variable: job satisfaction (*F*(5, 172): 20.139, *p* < 0.001); R^2^: 0.38					
Client incivility	‒0.017	0.031	‒0.034	‒0.551	0.583
Co‐worker incivility	0.048	0.030	0.118	1.579	0.116
Organisational support	**0.074**	**0.013**	**0.506**	**5.805**	**<0.001**
Team civility	**0.103**	**0.043**	**0.218**	**2.425**	**0.016**
Social support	‒0.003	0.028	‒0.009	‒0.119	0.905
Dependent variable: burnout (*F*(5, 172): 15.353, *p* < 0.001); R^2^: 0.32					
Client incivility	**0.100**	**0.025**	**0.259**	**3.979**	**<0.001**
Co‐worker incivility	0.019	0.024	0.061	0.775	0.439
Organisational support	**‒0.040**	**0.010**	**‒0.358**	**‒3.921**	**<0.001**
Team civility	‒0.039	0.034	‒0.107	‒1.134	0.258
Social support	‒0.004	0.022	‒0.013	‒0.166	0.868
Dependent variable: turnover intention (*F*(5, 167): 23.931, *p* < 0.001); R^2^: 0.43					
Client incivility	‒0.017	0.066	‒0.015	‒0.251	0.802
Co‐worker incivility	0.052	0.065	0.058	0.803	0.423
Organisational support	**‒0.121**	**0.028**	**‒0.373**	**‒4.357**	**<0.001**
Team civility	**‒0.237**	**0.091**	**‒0.227**	**‒2.608**	**0.010**
Social support	‒0.085	0.058	‒0.107	‒1.461	0.146
Dependent variable: stress (*F*(5, 172): 7.948, *p* < 0.001); R^2^: 0.19					
Client incivility	**0.046**	**0.021**	**0.156**	**2.212**	**0.028**
Co‐worker incivility	0.034	0.020	0.144	1.701	0.091
Organisational support	**‒0.022**	**0.009**	**‒0.258**	**‒2.595**	**0.010**
Team civility	‒0.001	0.028	‒0.004	‒0.042	0.966
Social support	‒0.014	0.018	‒0.068	‒0.784	0.434
Dependent variable: anxiety (*F*(5,167): 12.763, *p* < 0.001); R^2^: 28					
Client incivility	**0.296**	**0.112**	**0.178**	**2.643**	**0.009**
Co‐worker incivility	**0.325**	**0.108**	**0.241**	**3.003**	**0.003**
Organisational support	**‒0.140**	**0.046**	**‒0.292**	**‒3.080**	**0.002**
Team civility	0.095	0.151	0.061	0.631	0.529
Social support	‒0.133	0.099	‒0.113	‒1.341	0.182

Abbreviations: *B*, unstandardised coefficient; β, standardised coefficient; SE, standard error; *T*, *T*‐statistic

*Note*: Bold text signifies significant results.

Perceived organisational support and team civility were significant predictors of greater job satisfaction and lower turnover intentions. Perceived organisational support was also found to be a significant predictor of increased employee wellbeing, alongside burnout—which was linked to reduced employee wellbeing. Client incivility functioned as a predictor of higher levels of reported stress, alongside perceived organisational support—which was linked to lower employee stress levels. Finally, client incivility and co‐worker incivility were both significant predictors of higher levels of anxiety, alongside perceived organisational support—which was associated with lower levels of anxiety.

### R2: Organisational factors as mediators

To run a mediation analysis, the following criteria must be fulfilled: (a) the independent variable (incivility) must predict the dependent (anxiety) and the mediator (perceived organisational support), and (b) the mediator (perceived organisational support) must also predict the dependent variable (anxiety). Regression analysis found that client incivility did not significantly predict perceived organisational support. As such, a mediation analysis could only be conducted for the relationship between co‐worker incivility, organisational support and anxiety.

**FIGURE 1:**
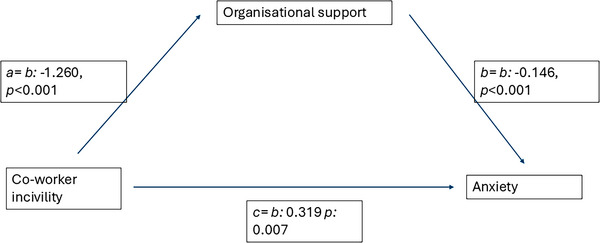
Mediation analysis indicating the model pathways (a‒c)

The mediation analysis was conducted in SPSS using the PROCESS macro, model number 4 (https://afhayes.com/introduction-to-mediation-moderation-and-conditional-processanalysis.html). The analysis assessed the mediating role of perceived organisational support on the relationship between co‐worker incivility and anxiety. The results revealed a significant relationship between co‐worker incivility and perceived organisational support (path a: ‒1.260 (0.223), *p* < 0.001). Co‐worker incivility also had a significant direct effect on anxiety (path c: 0.319 (0.116), *p* = 0.007), and perceived organisational support had a significant effect on anxiety (path b: ‒0.146 (0.041), *p* < 0.001), meaning that all paths in the model were significant. There was a significant indirect effect (path ab: 0.183 (0.0620), bootstrapping confidence interval 0.07–0.312). The direct effect of co‐worker incivility on anxiety in the presence of the mediator was also significant (total effect: 0.502 (0.109), *p* < 0.001). Combined, these results indicate a significant partial mediation, with co‐worker incivility having a direct impact on anxiety (path c) as well as an indirect impact via perceived organisational support (path ab). This mediation is competitive, in that paths a and b are negative and path c is positive. As such, perceived organisational support appears to reduce the effect of co‐worker rudeness on anxiety (see Figure 1).

### R3: Associations between responses to incivility, individual factors and organisational factors

A Pearson correlation matrix was developed (Table 5) to evaluate the potential associations between responses to incivility, individual factors and organisational factors. In the interest of brevity, only the key results will be reported in the text.

**TABLE 5:** Bivariate correlations between responses to client and co‐worker incivility with measures of individual factors (stress, anxiety, burnout, job satisfaction and turnover intention) and organisational factors (perceived organisational support, social support and team incivility)

Variables of interest	Friendly overture	Ignore	Confront	Exit	Avoid	Talk to friend	Involve colleague	Report	Emotion management	Consider reasons for behaviour
	Responses to client incivility									
Anxiety	0.109	0.109	‒0.018	0.016	**0.200** ^**^	**0.197** ^**^	0.040	0.086	0.107	0.015
Stress	‒0.001	0.095	0.048	‒0.039	0.137	0.115	‒0.021	‒0.013	0.052	‒0.055
Job satisfaction	‒0.002	‒0.087	0.066	‒0.077	**‒0.173** ^*^	‒0.139	0.003	‒0.012	‒0.083	0.084
Burnout	0.140	**0.242** ^**^	‒0.076	0.012	**0.188** ^*^	**0.165** ^*^	0.055	0.062	0.103	‒0.054
Turnover intention	0.112	**0.163** ^*^	‒0.072	0.037	0.143	0.122	‒0.052	‒0.039	0.026	‒0.047
Organisational support	‒0.087	**‒0.215** ^**^	0.107	0.023	**‒0.163** ^*^	**‒0.183** ^*^	0.016	0.136	0.019	**0.172** ^*^
Social support	0.012	‒0.124	0.129	‒0.091	**‒0.240** ^**^	0.022	0.006	**0.153** ^*^	‒0.019	**0.187** ^*^
Team civility	‒0.106	‒0.184	**0.217** ^**^	‒0.057	‒0.100	‒0.051	0.098	**0.153** ^*^	‒0.054	0.109
	Responses to co‐worker incivility									
Anxiety	**0.162** ^*^	**0.195** ^**^	‒0.025	0.077	**0.307** ^**^	0.103	0.006	0.047	0.023	0.028
Stress	0.115	0.133	0.059	0.010	**0.232** ^**^	0.095	‒0.036	‒0.002	0.061	0.031
Job satisfaction	0.075	‒0.020	0.042	‒0.098	**‒0.184** ^*^	‒0.130	0.017	‒0.046	0.090	**0.204** ^**^
Burnout	**0.160** ^*^	0.093	‒0.022	0.068	**0.260** ^**^	0.058	0.011	0.057	0.026	‒0.036
Turnover intention	0.021	‒0.001	‒0.013	0.131	**0.197** ^**^	0.082	‒0.095	‒0.029	‒0.099	**‒0.173** ^*^
Organisational support	‒0.021	0.090	0.106	‒0.130	**‒0.312** ^**^	**‒0.191** ^**^	0.058	0.029	0.122	**0.249** ^**^
Social support	0.079	‒0.041	0.102	‒0.033	**‒0.166** ^*^	0.043	0.111	0.091	0.086	**0.169** ^*^
Team civility	‒0.045	‒0.143	0.056	‒0.090	**‒0.245** ^**^	‒0.113	0.076	‒0.003	0.063	**0.247** ^**^

*Note*: Bold text signifies significant results, with asterisks indicating degree of significance.

^*^ *p* < 0.05.

^**^ *p* < 0.005.

The results for responses to client incivility indicate that six response types had significant correlations with employee and organisational factors. More specifically, ignoring, avoiding and talking to a friend/colleague were all positively correlated with burnout and negatively correlated with perceived organisational support. Avoid and talk to friend/colleague were also positively correlated with anxiety. Reporting and seeking underlying reasons for the behaviour were positively correlated with social support. Team civility correlated positively with both confrontable and reporting responses.

The findings for responses to co‐worker incivility indicate that six response types were associated with individual and organisational factors. Making a friendly overture, ignoring, avoidance and reciprocation were all positively correlated with anxiety. Reciprocation and avoidance were also negatively correlated with perceived organisational support and team civility climate. In contrast, seeking to understand the underlying reasons for the behaviour was positively correlated with perceived organisational support, social support and team civility climate.

### R4: Practice‐based strategies for managing incivility

Qualitative content analysis produced five key categories for each of the open‐ended questions (Table 6) regarding practice‐based strategies to manage incivility.

**TABLE 6:** Content analysis depicting categories of current and suggested veterinary practice strategies to manage incivility

Categories and codes for current practice strategies		Categories and codes for future suggested practice strategies	
Management via intervention by senior staff	Partners/practice management meet with instigator/target and discuss. Sack clients when necessary. Address the behaviour directly. Investigate and manage the incident. Issues raised with management. Senior staff monitor interactions and behaviours.	Management via intervention by senior staff	Take swift action. Sack clients when necessary. Leaders should avoid favouritism/clique development. Address underlying causes of incivility. Hold people accountable for their behaviour. Meet with the instigator/target. Conduct an external audit.
Talking about incivility	Open discussions. Practice meetings. Speaking up encouraged.	Listening and talking about incivility	Listen to concerns. Open discussions. Speaking up should be encouraged and supported. Group meetings to discuss and problem solve.
Knowledge and awareness	Education/training. Incivility champion. Posters. Incivility/civility materials.	Knowledge and awareness	Education/training. Raise client awareness of incivility/civility expectations. Encourage staff to reflect.
Supportive practice environment	Supportive practice environment. Generic support services (e.g., counselling).	Develop a supportive and civil practice environment	Support and care from leaders. Build a fair, supportive and civil culture. Leaders should model civility. Stress management. Encourage staff to reflect.
Lack of incivility policy or action	Nothing. Unclear policy.	Clear incivility policies	Clear policies. Zero tolerance policy.

### Current strategies for managing incivility

Methods for managing incivility included meetings with instigators and targets (management interventions by senior staff) or open discussions about incivility across the team (talking about incivility). Meetings were usually held on an individual basis, with a member of the senior management team instigating the discussion:

‘Line managers good at discussing situations and pulling aside person that is the problem’. P16

In contrast, when participants reported open discussion as a method, it was presented as a team‐based activity, with the aim being to openly exchange views and experiences:

‘We have had practice meetings, discussed and workshopped communication styles and personality types. We have a daily huddle where all voices are heard’. P52

Additional reported actions for managing incivility included de‐registering clients, reporting the behaviour and reprimanding the instigator.

Multiple participants reported that their practice did not have anything in place to address or manage incivility. In the majority of responses, it was suggested that incivility was simply ignored, there was a lack of protocol or that no action was taken:

‘There is not really any procedure in place, when issues are raised they are not acted on accordingly and are usually swept under the carpet’. P142

In some cases, participants indicated that nothing could be done because incivility issues originated with senior staff:

‘Nothing because the head vet is the main perpetrator of it’. P42

This could increase the difficulty of addressing the incivility and would lead to some strategies, such as senior staff meeting with the target of incivility, being inappropriate.

### Proposed future strategies for managing incivility

The participants suggested a range of proactive strategies to manage incivility in veterinary practices, including actions such as holding group meetings to discuss issues and engaging in problem solving to reach a solution. There were three key aspects within the data that appeared to be the underlying basis for many of the actions proposed: acting, listening and support.

The participants discussed the need for action to be taken, including investigations to determine the cause of the behaviour (management via intervention by senior staff), ideally soon after the uncivil incident has occurred:

‘Incivility should be addressed immediately as it happens’. P121

The participants also emphasised that staff should not be penalised for speaking up (listening and talking about incivility), and incivility should be acknowledged as subjective:

‘Listen with intent and process what you have heard, believe people when they share their lived experience … Don't victim blame, this is invalidating the person reporting, don't compare to others (we all experience situations differently)’. P15

Finally, many participants highlighted that support needed to be provided from the top down, with leaders setting the tone for the practice culture and approach to incivility (developing a supportive and civil practice environment):

‘Being approachable and a good listener. Feeling like there is someone who will support and stand up for you if need be’. P22

## DISCUSSION

The results replicate previous findings within the veterinary context,^12^ indicating that client incivility has a significant association with employee wellbeing. It also expands our understanding by suggesting that organisational support can mediate the impact of co‐worker incivility on anxiety. Organisational support and team civility were key predictors of job satisfaction and turnover intention, beyond workplace incivility. The findings also suggest that individual and organisational factors may be linked to the utilisation or selection of responses to incivility. Finally, qualitative data indicate the need for veterinary practices to address worker concerns, be proactive, provide clarity and enhance leadership support in managing incivility.

### Incivility, impact and organisational factors

Client incivility functioned as a predictor of veterinary staff anxiety, burnout and stress levels. There are two potential explanations for this finding within the literature. First, incivility is generally considered to have a detrimental impact on wellbeing through draining cognitive and emotional resources. Over time, this repeated reduction in resources leads to increasing levels of stress and exhaustion, as well as reducing the ability to recover.^25^ Second, the adverse impact of client incivility on wellbeing may be exacerbated through emotional labour, whereby staff feel obligated to present a professional and calm façade to customers, regardless of customer behaviour or employee internal emotions.^26^ Certainly, previous research has linked experiencing client incivility to emotional labour, with the suggestion that such labour is effortful and can lead to a range of adverse effects on wellbeing, including burnout.^26^

In our previous research, co‐worker incivility was linked to reduced job satisfaction and increased turnover intention.^6^ This result was initially replicated here, with co‐worker incivility positively correlated with turnover intention and negatively correlated with job satisfaction. However, the regression analysis found that organisational factors (perceived organisational support and team civility climate) proved to be more powerful predictors of job satisfaction and turnover intention. Previous research within the sphere of human healthcare mirrors this finding, with organisational support a key predictor of hospital employee^27^ job satisfaction. This relationship may be explained via organisational support theory, where support is suggested to enhance job satisfaction through meeting employee needs, indicating employee value and signalling that help is available.^28^ Organisational support may also buffer the impact of stressors (such as incivility) on employees through the provision of emotional and practical support, particularly in times of high work demand.^28^ Combined, these findings suggest that building organisational support via mechanisms such as fairness, supervisory support and autonomy^28^ has the potential to enhance job satisfaction, reduce turnover and buffer the impact of co‐worker incivility within veterinary practices.

### Factors linked to individual responses to incivility

The pattern of correlations between individual and organisational factors and reported responses to incivility was variable but suggested that there may be links between employee anxiety levels, perceived organisational support and the avoidance response, particularly in response to co‐worker incivility. The link between anxiety and avoidance strategies has been reported within the clinical literature, with the suggestion that individuals suffering from anxiety may attempt to avoid negative emotions and unpleasant situations.^29^ In contrast, organisational support may reduce the need for avoidance strategies via improved support, enhanced employee self‐efficacy and clarity around behavioural expectations.^28^ However, further research is needed to examine these potential associations.

### Practice‐based incivility management

Research within the veterinary setting has consistently indicated that staff consider a supportive practice environment essential in the management of incivility.^3^, ^6^ The current quantitative data build on this to suggest an association between team civility climate, social support and incivility reporting actions. As such, clarity in terms of civil behaviour expectations, as well as support from colleagues, could increase the likelihood of speaking up about incivility.

The current qualitative data highlight the importance of listening to staff concerns. Feeling heard, alongside being taken seriously, is an important aspect of organisational support and ensures that employees feel valued.^30^ Once concerns have been voiced, action should then be taken, ideally soon after the uncivil behaviour has occurred. The nature of suggested actions varied, including open or team discussions, one‐to‐one meetings with senior staff and taking part in educational or training activities. The educational approach has also been suggested within the human healthcare context.^31^ A combination of raising awareness, training staff to use different response strategies and implementing active learning appeared to improve nurses’ self‐efficacy and ability to manage incivility, but the results were variable across different forms of training.^31^ Further research is required within the veterinary setting, with interventions tailored to that context.

## LIMITATIONS

The data gathered here are subjective and may be influenced by participant memory and biases, including social desirability bias. The sample encompasses multiple veterinary staff roles but is predominantly reflective of veterinarians and veterinary nurses. The sample is also primarily female, so the findings may not be as applicable to male veterinary staff. The unbalanced nature of the sample (predominantly female veterinarians) meant that group comparisons could not be undertaken. The data are cross‐sectional, making causality more difficult to determine, and do not account for past experiences, including the potential influence of engagement with various response strategies over time.

## CONCLUSION AND RECOMMENDATIONS

The current study highlights the importance of organisational factors and support in the management of incivility within veterinary practices. These factors also appear important for maintaining job satisfaction and reducing quitting intention. More research is needed to develop and evaluate different forms of support and incivility interventions to identify best practice. In the meantime, the current study builds on previous research to produce the following suggested recommendations for veterinary practices:

- Listen to veterinary staff and take concerns regarding uncivil behaviour from clients and colleagues seriously.
- Try to manage incivility proactively by taking action through team discussions, one‐on‐one meetings with the staff involved and engaging in team training.
- Provide practical and emotional support to staff where possible, which may include the provision of clear guidelines for managing incivility and allowing time for staff to recover following an uncivil interaction.

## AUTHOR CONTRIBUTIONS

Amy Irwin, Luiz Santos, Helen Silver‐MacMahon and Liz Mossop contributed equally to the design and conception of the project. Amy Irwin created the SNAP survey and completed the required ethics processes. Amy Irwin, Luiz Santos, Helen Silver‐MacMahon and Liz Mossop engaged in data collection. Amy Irwin collated the data and conducted the analysis. Luiz Santos and Liz Mossop reviewed the analysis and the subsequent write‐up of the results section. Amy Irwin produced the initial draft of the paper. Luiz Santos, Helen Silver‐MacMahon and Liz Mossop reviewed and edited the paper, with Amy Irwin then producing the final draft.

## CONFLICT OF INTEREST STATEMENT

The authors declare no conflicts of interest.

## FUNDING INFORMATION

The authors received no specific funding for this work.

## ETHICS STATEMENT

This study was approved by the University of Aberdeen Psychology Ethics Committee (application id: 1061566).

## Data Availability

The data presented within this paper are not available publicly due to ethical and confidentiality constraints. The data will be shared by the corresponding author privately upon request.
